# Targeting surface-layer proteins with single-domain antibodies: a potential therapeutic approach against *Clostridium difficile*-associated disease

**DOI:** 10.1007/s00253-015-6594-1

**Published:** 2015-05-05

**Authors:** Hiba Kandalaft, Greg Hussack, Annie Aubry, Henk van Faassen, Yonghong Guan, Mehdi Arbabi-Ghahroudi, Roger MacKenzie, Susan M. Logan, Jamshid Tanha

**Affiliations:** Human Health Therapeutics Portfolio, National Research Council Canada, Ottawa, ON K1A 0R6 Canada; Department of Biology, Carleton University, Ottawa, ON K1S 5B6 Canada; School of Environmental Sciences, University of Guelph, Guelph, ON N1G 2W1 Canada; Department of Biochemistry, Microbiology and Immunology, University of Ottawa, Ottawa, ON K1N 6N5 Canada

**Keywords:** *Clostridium difficile*, Surface layer protein, Single-domain antibody, V_H_H, Nanobody

## Abstract

*Clostridium difficile* is a leading cause of death from gastrointestinal infections in North America. Antibiotic therapy is effective, but the high incidence of relapse and the rise in hypervirulent strains warrant the search for novel treatments. Surface layer proteins (SLPs) cover the entire *C. difficile* bacterial surface, are composed of high-molecular-weight (HMW) and low-molecular-weight (LMW) subunits, and mediate adherence to host cells. Passive and active immunization against SLPs has enhanced hamster survival, suggesting that antibody-mediated neutralization may be an effective therapeutic strategy. Here, we isolated a panel of SLP-specific single-domain antibodies (V_H_Hs) using an immune llama phage display library and SLPs isolated from *C. difficile* hypervirulent strain QCD-32g58 (027 ribotype) as a target antigen. Binding studies revealed a number of V_H_Hs that bound QCD-32g58 SLPs with high affinity (*K*_D_ = 3–6 nM) and targeted epitopes located on the LMW subunit of the SLP. The V_H_Hs demonstrated melting temperatures as high as 75 °C, and a few were resistant to the gastrointestinal protease pepsin at physiologically relevant concentrations. In addition, we demonstrated the binding specificity of the V_H_Hs to the major *C. difficile* ribotypes by whole cell ELISA, where all V_H_Hs were found to bind 001 and 027 ribotypes, and a subset of antibodies were found to be broadly cross-reactive in binding cells representative of 012, 017, 023, and 078 ribotypes. Finally, we showed that several of the V_H_Hs inhibited *C. difficile* QCD-32g58 motility in vitro. Targeting SLPs with V_H_Hs may be a viable therapeutic approach against *C. difficile*-associated disease.

**Electronic supplementary material:**

The online version of this article (doi:10.1007/s00253-015-6594-1) contains supplementary material, which is available to authorized users.

## Introduction

*Clostridium difficile* is currently the leading hospital-acquired infection in developed countries (Karas et al. 2010). As a Gram-positive, anaerobic, endospore-forming gastrointestinal (GI) pathogen, the bacterium causes *C. difficile*-associated disease (CDAD) in humans and animals. Symptoms of CDAD range from mild antibiotic-associated diarrhea to psuedomembraneous colitis and death, with an estimated associated health care cost of $3.2 billion annually in the USA (Dubberke and Olsen 2012; Ghantoji et al. 2010). From 2002 to 2005, the Canadian province of Québec suffered a CDAD epidemic, largely associated with a predominant strain referred to as North American pulsed-field type 1 (NAP1), ribotype 027, toxinotype III, and restriction endonuclease group BI (Bourgault et al. 2006; Gilca et al. 2010; Hubert et al. 2007; Loo et al. 2005; Pépin et al. 2004; Warny et al. 2005). These ribotype 027 strains were undetected in 2000 and 2001 but were responsible for the Québec outbreak in which its prevalence was estimated at 75.2 % of all polymerase chain reaction (PCR)-ribotyped strains in 2003 (MacCannell et al. 2006). Québec strain QCD-32g58 (NZ_CM000287.1) is one such isolate belonging to this group. Strains within PCR ribotype 027 have evolved to produce elevated levels of toxins A and B (Dupuy et al. 2008; Warny et al. 2005), have acquired antibiotic resistance cassettes (Bourgault et al. 2006; Pépin et al. 2005; Schmidt et al. 2007; Spigaglia et al. 2008; Stabler et al. 2009), and have shown enhanced sporulation ability (Åkerlund et al. 2008), all of which contribute to their virulence. Toxins A and B (TcdA and TcdB) are the primary *C. difficile* virulence factors and are therapeutic targets (Giannasca and Warny 2004; Hussack and Tanha 2010; Jank and Aktories 2008; Jank et al. 2007); however, targeting other virulence factors such as surface layer proteins (SLPs), cell wall proteins, and flagellar components have also been proposed as therapeutic strategies (Ghose 2013).

SLPs are common to almost all Archaea and can be found in nearly every phylogenetic group within Eubacteria (Fagan and Fairweather 2014; Sleytr and Beveridge 1999). These proteins have been identified as virulence factors for bacteria such as *Campylobacter fetus* and *Aeromonas salmonicida*, providing the cells with structural integrity, acting as molecular sieves and playing a role in adherence and immune evasion (Grogono-Thomas et al. 2000; Hamadeh et al. 1995; Sara and Sleytr 2000). *C. difficile* produces unique SLPs in that they are cleaved from a common precursor, SlpA, to generate the HMW and LMW subunits (Calabi et al. 2001). The two subunits associate to form mature proteins that cover the entire surface of the bacterium in a para-crystalline layer. The LMW subunit is highly immunogenic (Pantosti et al. 1989), is surface exposed (Fagan et al. 2009), and exhibits low inter-strain identity among different PCR ribotypes (Calabi and Fairweather 2002; Spigaglia et al. 2011). The high variability observed could be due to a lack of functional constraints or the evolutionary need to evade host immune responses. Indeed, *C. difficile* SLPs play a critical role in bacterial adherence to host cells (Calabi et al. 2002; Drudy et al. 2001; Merrigan et al. 2013; Takumi et al. 1991) and thereby contribute to colonization and the persistence of infection. They have also been shown to perturb cytokine homeostasis and modulate immune responses (Ausiello et al. 2006; Bianco et al. 2011; Collins et al. 2014; Ryan et al. 2011). SLPs induce maturation of dendritic cells and the subsequent generation of a T-helper cell response through Toll-like receptor 4 (TLR4), thereby altering host inflammatory and regulatory cytokines toward an inflammatory state and contributing to the damage of the intestinal epithelium. Interestingly, human patients with relapsing *C. difficile* incidences were found to exhibit a lower immunoglobulin M (IgM) response to SLPs compared to patients with a single *C. difficile* episode (Drudy et al. 2004), suggesting that the ability to mount an anti-SLP antibody response may significantly determine a patient’s disease state. Collectively, these studies support the hypothesis of an important role for SLPs in innate and adaptive immunity.

A limited number of examples suggest targeting SLPs could be a potential therapeutic approach to combat CDAD. O’Brien et al. (2005) demonstrated that prophylactic administration of SLP anti-sera significantly prolonged survival of hamsters that were lethally challenged. Subsequent studies of active immunization of mice using crude cell wall extracts showed a significant reduction in *C. difficile* colonization of the immunized group compared to controls (Péchiné et al. 2007). Currently, *C. difficile* infections are treated with a course of antibiotics, which can alter the composition of the gut microbiome and increase the selection pressure on the organism, which can in turn lead to antibiotic resistance. Targeting essential bacterial virulence factors, such as SLPs, is an alternative therapeutic strategy to conventional antibiotic use, which can address the risk of rising antibiotic resistance (Cegelski et al. 2008; Clatworthy et al. 2007; Lynch and Wiener-Kronish 2008).

Single-domain antibodies isolated from the variable domains of Camelidae species heavy-chain IgGs (referred to as V_H_Hs or “Nanobodies”) are attractive candidates to explore for oral therapy because these domains retain the affinity and specificity of conventional monoclonal antibodies (mAbs), but possess added biophysical advantages such as resistance to extreme pH and proteases (Harmsen and De Haard 2007; Holliger and Hudson 2005; Holt et al. 2003). Single-domain antibodies have been isolated to many targets in the context of infection and immunity (Hussack and Tanha 2010; Wesolowski et al. 2009), and their potential as oral therapeutics has been well documented (Harmsen et al. 2007; van der Vaart et al. 2006; Virdi et al. 2013).

The use of antibodies as neutralizing agents, in addition to studies implicating *C. difficile* SLPs as mediators for cell-host interactions (Calabi et al. 2002; Drudy et al. 2001), has inspired the current study. Here, V_H_Hs to SLPs from *C. difficile* strain QCD-32g58 were selected from an immune llama V_H_H phage display library. The antibodies were then functionally and biochemically characterized with respect to structure, affinity, specificity, aggregation state, thermostability, resistance to pepsin digestion, and the ability to bind and inhibit the motility of *C. difficile* cells.

## Materials and methods

### Isolation of SLPs from *C. difficile* strains 630 and QCD-32g58

*C. difficile* SLPs were isolated using low-pH glycine extraction as described previously (Dubreuil et al. 1988) with the following modifications. Briefly, cells from strains QCD-32g58 (GenBank accession no. AAML00000000; Janvilisri et al. 2009; Forgetta et al. 2011) and 630 (GenBank accession no. AM180355.1; Janvilisri et al. 2009; Monot et al. 2011; Sebaihia et al. 2006) were cultured overnight on a BHI-agar plate, scraped, resuspended in 500 μl of 0.2 M glycine, pH 2.2, and incubated for 10 min at room temperature. Bacterial cells were removed by centrifugation at 13,000 rpm in a benchtop centrifuge and the SLP-containing supernatant transferred to a 4-ml Amicon filter device with a 5000 Da MWCO (EMD Millipore, Toronto, ON, Canada) for buffer exchange. The SLPs were washed twice with 4 ml of sterile H_2_O and collected in 1 ml sterile H_2_O. A 10-μl aliquot was mixed with SDS-PAGE loading buffer containing β-mercaptoethanol and analyzed on a 12.5 % SDS-PAGE gel. Size-exclusion chromatography (SEC) was used to further purify the isolated SLP proteins after extraction. To this end, a Superdex™ 200 10/30 GL column (GE Healthcare, Baie-d’Urfé, QC, Canada) was equilibrated with running buffer (10 mM HEPES buffer, pH 7.5, 150 mM NaCl), and 500 μl of SLP extracts were loaded and eluted over one column volume as previously described (Fagan et al. 2009). Eluted fractions were analyzed on a 12.5 % SDS-PAGE for content. All fractions were stored at 4 °C for later use.

### Llama immunization, V_H_H phage display library construction, and panning

Llama immunization, library construction, and panning were carried out as described previously (Hussack et al. 2012). Briefly, for llama immunization, one adult male llama (*Lama glama*) was immunized subcutaneously four times at its lower back with a mixture of QCD-32g58 and 630 SLP antigens at the Cedarlane animal facility (Burlington, ON, Canada) and according to the company’s animal safety protocol. On the first day, a pre-immune bleed was conducted and a mixture of two antigens (100 μg of each antigen diluted in PBS in total volume of 1.25 ml) and Freund’s complete adjuvant (1.25 ml; Sigma, Oakville, ON, Canada) was injected into the llama. The llama received three additional boosts with 100 μg of the same antigen mixture with Freund’s incomplete adjuvant (Sigma) on days 28, 47, and 66. Blood (10–15 ml) was collected on days 59 and 72. Total (un-fractionated) serum was analyzed for a specific response to QCD-32g58 and 630 SLPs by enzyme-linked immunosorbent assay (ELISA). Llama serum from day 72 was fractionated into conventional (IgG1) and heavy-chain antibody (IgG2 and IgG3) components and analyzed for specific binding to QCD-32g58 and 630 SLPs by ELISA (Hussack et al. 2012). Lymphocytes were isolated at Cedarlane. A V_H_H phage display library was constructed using approximately 2 × 10^7^ lymphocytes (as the source of V_H_H repertoire genes) collected from the day 72 blood. The size of the library was estimated to be 2.7 × 10^8^ transformants. The V_H_H DNA fragments from 92 colonies were PCR-amplified and sequenced to assess library diversity. Library phage was prepared and 10^12^ colony-forming units (CFU) of library phage was used as input for the first round of panning against 10 μg of SEC-purified QCD-32g58 SLPs coated onto NuncMaxisorp™ wells (Thermo Fisher, Ottawa, ON, Canada). For the following three rounds of panning, 10^11^ CFU phage was used as the input. Phage ELISA was performed to identify individual phage displaying V_H_Hs specific to QCD-32g58 and 630.

### V_H_H subcloning, soluble expression, purification, and SEC

Positive V_H_H binders identified by phage ELISA were subcloned, expressed in 1-l cultures and purified by immobilized metal-ion affinity chromatography as described (Hussack et al. 2012). Purified proteins were assessed for purity and integrity by SDS-PAGE. The aggregation status and elution volumes of V_H_Hs were determined by SEC using a Superdex™ 75 10/300 GL column (GE Healthcare) as described (Hussack et al. 2012; Kim et al. 2012a). Elution volumes were used to determine apparent molecular masses (*M*_app_s) of V_H_Hs from a set of protein standards (Hussack et al. 2011b). SEC chromatograms were normalized as described (Kim et al. 2012b).

### SPR analysis

The binding of all V_H_Hs to immobilized QCD-32g58 SLP, 630 SLP, and QCD-32g58 SLP LMW subunit was determined by surface plasmon resonance (SPR) using a Biacore 3000 (GE Healthcare). The SLPs were SEC-purified as described above prior to immobilization at concentrations of 50 μg/ml in 10 mM acetate buffer on a CM5 sensor chip using the amine coupling kit supplied by the manufacturer (GE Healthcare). In all instances, analyses were carried out at 25 °C in 10 mM HEPES running buffer, pH 7.4, containing 150 mM NaCl, 3 mM EDTA, and 0.005 % surfactant P20 at a flow rate of 20 μl/min. For regeneration, the surfaces were washed thoroughly with either running buffer (SLP_V_H_H2, SLP_V_H_H26, SLP_V_H_H49, and SLP_V_H_H50), 10 mM glycine-HCl, pH 3.0, for 3 s (SLP_V_H_H22), 10 mM glycine-HCl, pH 2.5, for 3 s (SLP_V_H_H5 and SLP_V_H_H46), or 50 mM NaOH for 3 s (SLP_V_H_H12 and SLP_V_H_H23). Due to the loss of surface activity after 50 mM NaOH surface regeneration, a fresh surface was made and used to study the binding activity of SLP_V_H_H12 and SLP_V_H_H23. Data were analyzed with BIAevaluation 4.1 software.

### *T*_m_ measurements by circular dichroism spectroscopy

The thermal unfolding profile of each antibody was obtained using circular dichroism (CD) according to a previously described method (Hussack et al. 2011b) with minor modifications. Briefly, after dialysis into 10 mM sodium phosphate buffer, pH 7.0, a 1-mm cuvette containing 200 μl of a V_H_H at 50 μg/ml was used to obtain CD spectra from 180–260 nm using a J-810 spectropolarimeter (Jasco Inc., Easton, MD, USA). The temperature was increased from 30 to 96 °C at a temperature ramp rate of 1 °C/min, and data were collected every 2 °C at a spectral scan rate of 50 nm/min and 1-mm bandwidth.

### Disulfide bond mapping by MS

Disulfide bond mapping of SLP_V_H_H22 and SLP_V_H_H50, each with four Cys residues, was performed essentially as described (Kim et al. 2012b; Hussack et al. 2011b). Briefly, tryptic fragments for subsequent mass spectrometry (MS) analysis were prepared as described (Kim et al. 2012a). Aliquots of V_H_H proteolytic digests were resuspended in 0.1 % (*v*/*v*) formic acid (aq) and analyzed by nanoflow reversed-phase HPLC MS (nanoRPLC-ESI-MS) with data-dependent analysis (DDA) using collision-induced dissociation (CID) on a nanoAcquity UPLC system coupled to a Q-TOF Ultima™ hybrid quadrupole/TOF mass spectrometer (Waters, Milford, MA, USA). The peptides were first loaded onto a 300 μm I.D. × 5 mm C18 PepMap100 μ-precolumn (Thermo Fisher) and then eluted into a 100 μm I.D. × 10 cm 1.7-μm BEH130C18 column (Waters) using a linear gradient from 0 to 36 % solvent B (acetonitrile + 0.1 % formic acid) over 36 min followed by 36–90 % solvent B for 2 min. Solvent A was 0.1 % formic acid in water. The peptide MS^2^ spectra were compared with V_H_H protein sequences using the Mascot™ database searching algorithm (Matrix Science, London, UK). The MS^2^ spectra of the disulfide-linked peptides were de-convoluted using the MaxEnt 3 program (Waters) for de novo sequencing to confirm and/or determine the exact disulfide linkage positions.

### Pepsin digestion assay

To assess the degree of resistance of each antibody to pepsin (a common protease in the digestive tract), SLP-specific V_H_Hs were subjected to pepsin digestion as previously described (Hussack et al. 2011b) at enzyme concentrations ranging from 1.25 to 100 μg/ml. Triplicate independent experiments were conducted, and densitometry analysis values were averaged to determine percent pepsin resistance.

### Epitope characterization by Western blot analysis

To determine subunit specificity of the V_H_Hs and the nature of their epitope (conformational or linear), denaturing Western blots of strain QCD-32g58 SLPs were probed with anti-SLP V_H_Hs. SLPs (5 μg/lane) were separated on 12.5 % SDS-PAGE gels and transferred to PVDF membranes at 100 V for 1 h. Membranes were blocked for 2 h with 2 % (*w*/*v*) milk in PBS and probed with various V_H_Hs (50 μg/5 ml PBS-T [PBS/0.05 % (*v*/*v*) Tween 20]) for 1 h. Membranes were washed three times in PBS-T followed by addition of mouse anti-His IgG-alkaline phosphatase (AP) conjugate (Abcam, Cambridge, MA, USA), diluted 1:5000 in blocking buffer, for 1 h. Membranes were washed as before and subjected to AP substrate (Bio-Rad, Mississauga, ON, Canada) for 10 min, washed in distilled H_2_O and air dried. A corresponding stained SDS-PAGE gel of the SLPs was used as reference.

### Whole cell ELISA

*C. difficile* strains were grown on BHI supplemented agar under anaerobic conditions at 37 °C overnight. Cells were resuspended in PBS containing 3 % (*v*/*v*) formalin and left for 24 h at 4 °C. Cells were washed two times with PBS and resuspended to OD_600_ 0.08. NuncMaxiSorp® Flat-Bottom plates were coated with 100 μl of formalin-fixed cells overnight at 37 °C. Plates were blocked with 2 % (*w*/*v*) milk in PBS. His_6_-tagged V_H_Hs specific for SLP were then added (10 μg/ml in PBS-T) and plates incubated at 37 °C for 1 h in a shaker incubator. Plates were washed three times with PBS-T and then incubated with rabbit anti-His_6_ antibody-horse radish peroxidase conjugate (1:5,000 in PBS-T, of a 1 mg/ml stock; Cedarlane) for 1 h at 37 °C. Following washing as above, the antibody was detected with TMB substrate for 10 min and the reaction stopped with 1 M H_3_PO_4_. Samples were analyzed in triplicate, and the absorbance was measured at 450 nm.

### Motility assay

An in vitro motility assay was used to determine if the isolated V_H_Hs were capable of binding whole *C. difficile* cells and preventing motility. Sterile culture tubes containing 5 ml 0.175 % agar-BHI media supplemented with 0.5 % (*w*/*v*) Bacto-yeast extract, 0.12 % (*w*/*v*) NaCl, and 25 or 50 μg/ml V_H_H, were stabbed with a fresh culture of strain QCD-32g58 as previously described (Twine et al. 2009) and incubated in anaerobic conditions at 37 °C for 23 h. Photographs were taken at 23 h postinoculation to monitor the effects of each antibody on motility of the strain relative to a control which did not receive antibody.

## Results

### Purification of SLPs from 630 and QCD-32g58 *C. difficile* strains

**Fig. 1:**
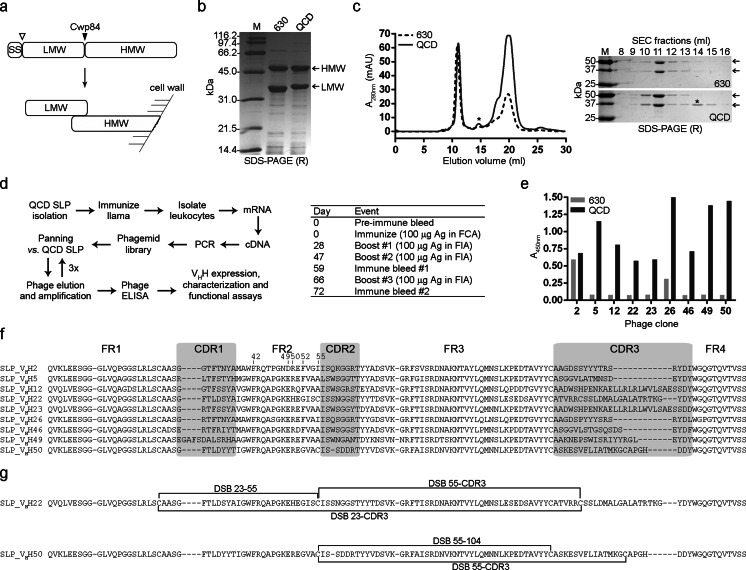
Isolation of SLP-specific V_H_Hs. **a** Schematic diagram of *C. difficile* S-layer proteins. *Top*, SLP low-molecular-weight (LMW) and high-molecular-weight (HMW) subunits are expressed as a single polypeptide chain before cleavage with Cwp84 cysteine protease. The cleavage site of the signal sequence (SS) is also shown. *Bottom*, after Cwp84-mediated cleavage, the LMW and HMW subunits associate in the orientation relative to the bacterial cell wall shown. **b** SDS-PAGE, run under reducing (R) conditions, of SLPs purified from 630 and QCD-32g58 (QCD) strains using low pH extraction. **c** *Left*, SEC Superdex™ 200 profile of SLPs and, *right*, reducing SDS-PAGE gel of the corresponding fractions. Only LMW subunit from QCD-32g58 could be purified (shown with an *asterisk*). The HMW subunit could not be purified from either strain. **d** Work flow overview and llama immunization schedule for the isolation of SLP-specific V_H_Hs. *FCA* Freund’s complete adjuvant, *FIA* Freund’s incomplete adjuvant, *Ag* QCD-32g58 SLP. **e** Phage ELISA demonstrating the binding of phage-displayed V_H_Hs to immobilized SLPs. **f** Amino acid sequence alignment of V_H_Hs isolated from panning that were expressed and characterized in this study. Positions 42, 49, 50, 52, and 55 are numbered. Numbering and CDR designations are according to IMGT (http://imgt.cines.fr/). **g** Unusual disulfide bonds (DSB) identified in SLP_V_H_H22 and SLP_V_H_H50 by mass spectrometry fingerprinting analysis

SLPs from *C. difficile* strains 630 and QCD-32g58 (Figs. 1a and S1) were first purified by low pH glycine extraction. When analyzed by reducing SDS-PAGE, the HMW and LMW SLPs migrated to ~45 and ~33 kDa (630) and ~45 and ~34 kDa (QCD-32g58) (Fig. 1b), which is close to the predicted *M*s of 39.5/34.2 kDa and 44.2/33.9 kDa (HMW/LMW SLPs, from 630 and QCD-32g58 strains, respectively) and consistent with others who ran SLPs under reducing SDS-PAGE conditions (Calabi et al. 2001; Mauri et al. 1999). To increase SLP purity, low pH extracted-SLP preparations were injected over a Superdex™ 200 SEC column (Fig. 1c, left panel). Fractions from the two major peaks and one minor peak were analyzed by 12.5 % SDS-PAGE (Fig. 1c, right panel). The first peak (with an elution volume of 10.8 ml), when analyzed by SDS-PAGE, confirmed the presence of both HMW and LMW subunits of SLPs. The second minor peak eluting at approximately 15 ml corresponded to the LMW subunit. The LMW subunit could only be isolated from QCD-32g58. The last major SEC peak was not detectable on SDS-PAGE despite the strong A_280nm_ signal, which could represent breakdown products of the HMW subunit, as it is unstable once separated from the LMW subunit (Fagan et al. 2009), and since the HMW subunit was not isolated in free-form from any of the fractions collected. The SEC-purified QCD-32g58 SLP and LMW SLP were used in library panning and SPR experiments.

### Llama immunization, library construction, and panning for SLP-binding V_H_Hs

V_H_Hs isolated from naive libraries tend to have low target antigen affinities (*K*_D_s in the μM range; Tanha et al. 2002; Yau et al. 2005); therefore, an immune llama library was constructed to isolate high affinity binders to SLPs, using a mixture of 630 and QCD-32g58 SLPs as immunogens. A male llama was immunized using an equal mixture of both antigens, according to the schedule in Fig. 1d. Llama sera and blood were processed and a heavy-chain IgG response to QCD-32g58 SLP was determined by ELISA (data not shown). An immune phage display library was constructed and was subjected to four rounds of panning against SLPs from QCD-32g58 (Fig. 1d). To identify QCD-32g58-specific binders after three rounds of panning, a total of 50 TG1 *E. coli* colonies containing the phagemid vector were picked at random for monoclonal phage ELISA to identify binders to QCD-32g58 SLP (data not shown). Nine unique V_H_Hs were identified, and the phage ELISA is shown for those clones (Fig. 1e). The amino acid sequence composition of the nine unique antibodies (Fig. 1f) confirmed their identity as V_H_Hs (not V_H_s), according to characteristic camelid V_H_H residues at positions 42, 49, 50, and 52 (Harmsen et al. 2000). The V_H_Hs were denoted SLP_V_H_H2, SLP_V_H_H5, SLP_V_H_H12, SLP_V_H_H22, SLP_V_H_H23, SLP_V_H_H26, SLP_V_H_H46, SLP_V_H_H49, and SLP_V_H_H50 (Fig. 1f). Based on the phage ELISA (Fig. 1e), all nine clones showed specific binding to SLP from QCD-32g58 while only SLP_V_H_H2, and less strongly SLP_V_H_H26, cross-reacted to SLP from strain 630. This is not surprising as the V_H_H library was panned against QCD-32g58. The CDR3 length distribution among the nine antibodies isolated varied. SLP_V_H_H2, SLP_V_H_H5, and SLP_V_H_H26 have the shortest CDR3 with 16 residues. SLP_V_H_H12, SLP_V_H_H22, and SLP_V_H_H23 all have a significantly long CDR3, with lengths of 28, 25, and 28 residues, respectively. Many of the clones shared high sequence homology, while SLP_V_H_H22 and SLP_V_H_H50 contained an additional cysteine residues at position 55 and in complementarity-determining region 3 (CDR3). The presence of a cysteine at residue 55 is characteristic of V_H_H subfamilies 3 and 4 (Harmsen et al. 2000). These two V_H_Hs were the only binders to belong to the V_H_H subfamily 3 while the other V_H_Hs were subfamily 1. Cys55 and CDR3 Cys have the potential to form an interloop disulfide bond to restrict the fold of the relatively long CDR3 and enhance the stability of the antibodies (Govaert et al. 2012; Kim et al. 2014). This indeed was shown to be the case for both SLP_V_H_H22 and SLP_V_H_H50 by MS-based disulfide bond mapping experiments (Fig. 1g; Table S1). However, and unexpectedly, disulfide bond mapping also revealed that these noncanonical Cys residues were also involved in forming other, unusual disulfide linkages. In SLP_V_H_H22, Cys55 and CDR3 Cys form disulfide linkages with Cys23, which typically forms a highly conserved disulfide linkage with Cys104 in V_H_Hs, and similarly in SLP_V_H_H50, Cys55 forms a disulfide linkage with Cys104.

### Expression and biophysical characterization of SLP-binding V_H_Hs

The nine SLP-binding V_H_Hs isolated from panning were subcloned, expressed, and purified. We observed high and variable expression yields of the clones (15–75 mg/l of bacterial culture). Purified V_H_Hs were subjected to SEC analysis, and all were nonaggregating monomers as expected, with a mean ± SD *M*_app_ of 15.9 ± 2.4, similar to the mean ± SD theoretical mass of 16.3 ± 0.6 expected for monomeric V_H_Hs (Fig. S2a; Table 1). We further characterized the panel of V_H_Hs by determining midpoint unfolding temperatures (*T*_m_s) by CD spectroscopy and V_H_H sensitivities to the major gastrointestinal enzyme pepsin by proteolytic digestion assays. Both techniques provide valuable information on V_H_H stability and aid in the selection of lead candidates. From the heat-induced unfolding curves, the V_H_H *T*_m_s ranged from 62.3 to 75.4 °C (Fig. S2b; Table 1) with all V_H_Hs folded at physiological temperatures. Antibody unfolding followed a single phase transition as expected. Next, all V_H_Hs were subjected to a pepsin digestion assay at pH 2.0, beginning with a biologically relevant concentration of pepsin at 100 μg/ml (Fig. S3). Under digestion conditions, the V_H_Hs exhibited a loss of the C-terminal tag, consistent with our previous findings (Hussack et al. 2011b; To et al. 2005), and therefore lower bands corresponding to a *M* that is ~2 kDa less than the band corresponding to the full-length V_H_H are considered as resistant to enzymatic digestion. As expected, resistance to pepsin decreased as a function of enzyme concentration (Table 1; Fig. S3). High pepsin resistance was observed at lower pepsin concentrations and the majority of V_H_Hs (five out of nine) showed moderate to high resistance at 25 μg/ml pepsin concentration. SLP_V_H_H2 and SLP_V_H_H22 showed the greatest pepsin resistance with an average of 12 ± 3.1 % and 19.6 ± 0.8 % V_H_H remaining after digestion for 1 h with 100 μg/ml of enzyme, respectively (Table 1). At lower pepsin concentrations (50 μg/ml), 15.3 ± 5.0 % of SLP_V_H_H2, 46.5 ± 10.0 % of SLP_V_H_H22, 21.9 ± 9.8 % of SLP_V_H_H23 and 2.8 ± 2.0 % of SLP_V_H_H12 remained undigested after 1 h.

**Table 1:** Summary of V_H_H molecular mass, thermal stability, and pepsin resistance data

V_H_H	*M* (kDa)	*M*_*app*_ (kDa)	*T*_m_ (°C)	Pepsin resistance (%)^a^		
				100 μg/ml	10 μg/ml	1.25 μg/ml
SLP_V_H_H2	15.71	14.5	62.3	12.0 ± 3.1	55.3 ± 13.1	99.0 ± 1.3
SLP_V_H_H5	15.61	14.2	70.3	0	10.3 ± 1.5	76.1 ± 15
SLP_V_H_H12	17.00	16.6	73.7	0	77.8 ± 3.9	99.4 ± 1.9
SLP_V_H_H22	16.38	17.3	74.6	19.6 ± 0.8	83.1 ± 3.3	99.0 ± 1.5
SLP_V_H_H23	17.02	19.1	75.4	0	93.4 ± 5.9	97.2 ± 1.7
SLP_V_H_H26	15.72	14.2	71.9	0	50.8 ± 2.5	96.6 ± 0.1
SLP_V_H_H46	15.83	16.6	66.3	0	55.6 ± 4.5	96.6 ± 1.6
SLP_V_H_H49	16.71	11.9	64.8	0	0	59.7 ± 14.2
SLP-V_H_H50	16.25	18.7	70.3	0	15.9 ± 7.9	89.9 ± 3.1

*M* theoretical (formula) molecular mass, *M*_app_ apparent molecular mass determined by SEC, *T*_m_ melting temperature

^a^Percent V_H_H (mean ± SE) remaining after digestion for 1 h at 37 °C and pH 2.0 with 100, 10, or 1.25 μg/ml of pepsin (*n* = 3)

### Binding analysis of V_H_Hs to SLPs

**Fig. 2:**
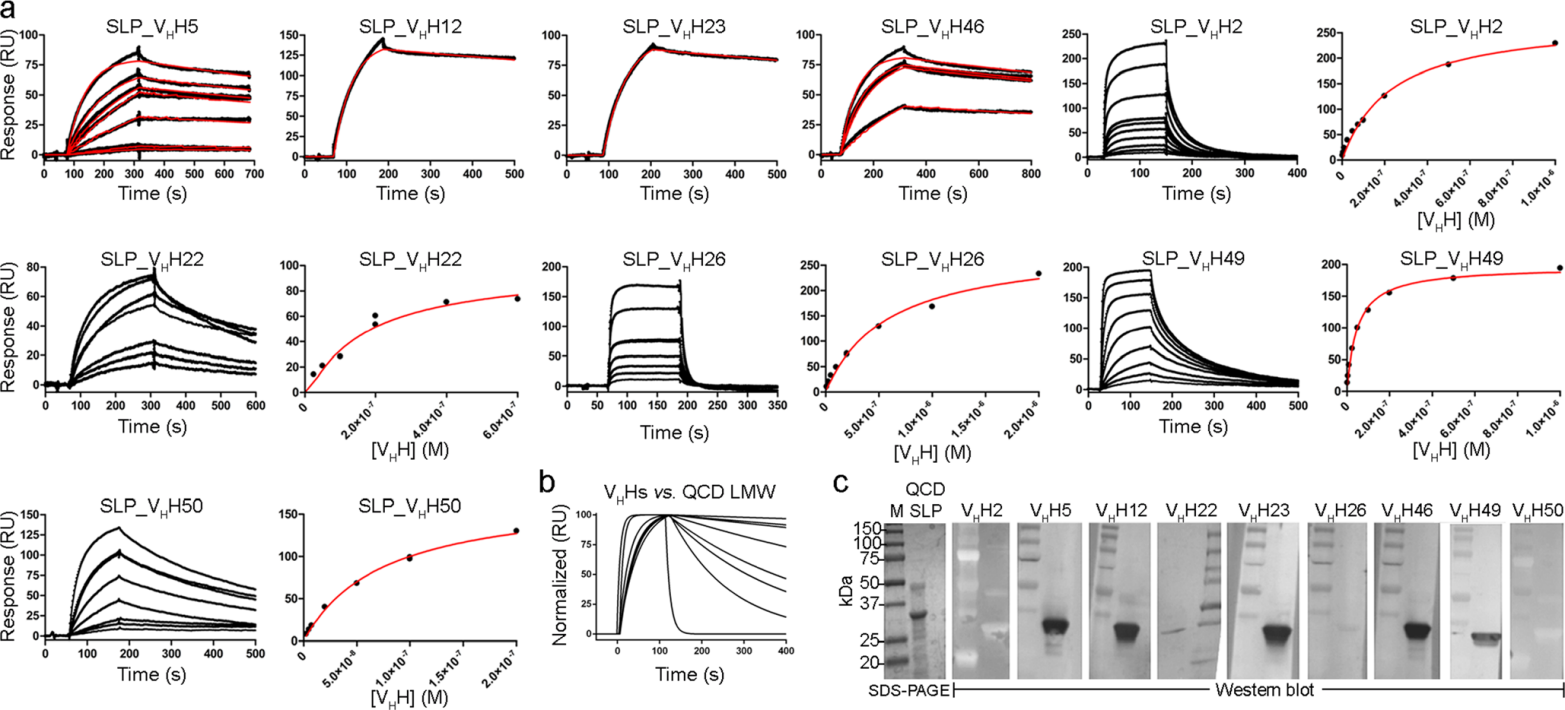
Characterization of V_H_H binding to SLPs. **a**, **b** SPR sensorgrams illustrating the binding of V_H_Hs to immobilized QCD-32g58 SLP (**a**) and QCD-32g58 LMW SLP (**b**). **c** Western blots demonstrating that a subset of V_H_Hs recognizes a liner epitope on the LMW subunit of QCD-32g58 SLP. *QCD* QCD-32g58

For affinity determination, monomeric fractions of V_H_Hs collected from the SEC column were analyzed by SPR. V_H_Hs were injected over CM5-immobilized and SEC-purified QCD-32g58 SLP, 630 SLP, and the QCD-32g58 LMW subunit, at various concentrations to characterize the binding specificity and affinity (Fig. 2a, b). In the first experiment, all nine V_H_Hs were shown to bind QCD-32g58 SLP (Fig. 2a; Table 2). None of the V_H_Hs bound to the reference surface on which a similar amount of a control protein was immobilized (data not shown). *K*_D_s were determined from kinetic rate constants (SLP_V_H_H5, SLP_V_H_H12, SLP_V_H_H23, and SLP_V_H_H46) or by steady-state analysis (SLP_V_H_H2, SLP_V_H_H22, SLP_V_H_H26, SLP_V_H_H49, and SLP_V_H_H50). The V_H_Hs SLP_V_H_H5, SLP_V_H_H12, SLP_V_H_H23, and SLP_V_H_H46 had the highest affinities to QCD-32g58 SLP (*K*_D_s of 3–6 nM). SLP_V_H_H12 and SLP_V_H_H23 required the use of 50 mM NaOH for their complete dissociation from the QCD-32g58 SLP surface, which resulted in loss of surface activity; therefore, a fresh surface was made, and only a single injection of each was used to analyze the binding activity of these two V_H_Hs. SLP_V_H_H49 and SLP_V_H_H50 had affinities of 48 and 75 nM, respectively. SLP_V_H_H2, SLP_V_H_H22, and SLP_V_H_H26 had the weakest affinities to QCD-32g58 SLP with *K*_D_s of 230, 180, and 580 nM, respectively. These three V_H_Hs, as well as SLP_V_H_H49, showed a complex binding pattern to QCD-32g58 SLP in that at low antibody concentrations, high-affinity binding was observed, while at high antibody concentrations lower affinity binding was observed, which maybe an indicator of antigen heterogeneity. Collectively, the SPR data confirmed the ability of the V_H_Hs to bind QCD-32g58 SLP.

**Table 2:** SLP-specific V_H_H binding data

V_H_H	QCD-32g58 SLP				QCD-32g58 LMW SLP^a^			
	*k*_on_ (/M/s)	*k*_off_ (/s)	*K*_D_ (nM)	R_max_ (RU)	*k*_on_ (/M/s)	*k*_off_ (/s)	*K*_D_ (nM)	R_max_ (RU)
SLP_V_H_H2	n.d.^b^	n.d.^b^	230	277	1.5 × 10^5^	1.3 × 10^−2^	90	26
SLP_V_H_H5	8.2 × 10^4^	4.6 × 10^−4^	6	100	1.4 × 10^5^	4.1 × 10^−4^	3	151
SLP_V_H_H12	1.2 × 10^5^	3.4 × 10^−4^	3	142	1.4 × 10^5^	1.2 × 10^−4^	1	131
SLP_V_H_H22	n.d.^b^	n.d.^b^	180	100	1.3 × 10^5^	1.1 × 10^−3^	8	114
SLP_V_H_H23	9.4 × 10^4^	3.7 × 10^−4^	4	98	1.1 × 10^5^	3.2 × 10^−4^	3	72
SLP_V_H_H26	n.d.^b^	n.d.^b^	580	288	2.1 × 10^5c^	9.7 × 10^−2c^	460^c^	5^c^
SLP_V_H_H46	1.1 × 10^5^	3.4 × 10^−4^	3	83	1.5 × 10^5^	3.2 × 10^−4^	2	181
SLP_V_H_H49	n.d.^b^	n.d.^b^	48	197	5.9 × 10^5^	1.2 × 10^−2^	20	231
SLP-V_H_H50	n.d.^b^	n.d.^b^	75	175	1.9 × 10^5^	2.7 × 10^−3^	14	154

^a^Binding kinetics were determined from 200 nM V_H_H injections as a binding screen

^b^A steady-state model was used to obtain the *K*_D_. Therefore, rate constants are not determined (n.d.)

^c^The affinity and rate constants should be interpreted with caution as the experimental R_max_ is very low, and multiple injection are required to confirm the values

Next, we expanded our SPR analyses to determine if the V_H_Hs cross-reacted to 630 SLP. In a similar approach to the QCD-32g58 SLP, 630 SLP were immobilized on a CM5 sensor chip and V_H_Hs injected at various concentrations. Consistent with our earlier phage ELISA results (Fig. 1e), only SLP_V_H_H2 and SLP_V_H_H26 bound 630 SLP (data not shown). The affinities of SLP_V_H_H2 and SLP_V_H_H26 to 630 SLP were 1 and 2 μM, respectively, indicating a ~5-fold weaker binding affinity to 630 SLP than QCD-32g58 SLP.

Finally, we set out to explore the nature of the QCD-32g58 SLP epitope recognized by the V_H_Hs, specifically if they bound the HMW or LMW SLP subunit. As previously shown (Fig. 1c), we were unable to purify the HMW SLP subunit and purified only a small amount of the QCD-32g58 LMW SLP subunit which limited our SPR analysis against the LMW SLP to a single concentration screen. At 200 nM V_H_H concentrations, all of our V_H_Hs bound the QCD-32g58 LMW subunit (Fig. 2b; Table 2). A similar affinity rank pattern to the full SLP was observed: SLP_V_H_H5, SLP_V_H_H12, SLP_V_H_H23, and SLP_V_H_H46 had the lowest *K*_D_s of all V_H_Hs tested, SLP_V_H_H2 and SLP_V_H_H26 had the highest *K*_D_s, and the remaining V_H_Hs had intermediate *K*_D_s. Interestingly, the V_H_Hs bound with higher affinities to the LMW SLP than the full SLP, suggesting a more optimal epitope presentation on the SPR chip for the LMW SLP. Collectively, the SPR binding data indicated the epitopes recognized by anti-SLP V_H_Hs reside entirely in the LMW subunit of QCD-32g58 SLP, and that some level of cross-reactivity to 630 SLP, presumably with the LMW subunit, was evident for a subset of the V_H_Hs. These findings are consistent with earlier reports that showed the LMW SLP subunit is immunodominant (Spigaglia et al. 2011) and that cross-reactive antibodies to the LMW SLP subunit from different *C. difficile* ribotypes are rare due to the low amino acid sequence homology (Calabi et al. 2001). To determine if the QCD-32g58 SLP epitope recognized by the V_H_Hs was linear or conformational, a denaturing SDS-PAGE-Western blot was performed. QCD-32g58 SLPs were separated in an SDS-PAGE gel under reducing conditions, transferred to a PVDF membrane, and probed with individual V_H_Hs followed by detection with an anti-His_6_ IgG conjugated to alkaline phosphatase (Fig. 2c). A nontransferred SDS-PAGE was run to demonstrate the presence of both HMW and LMW QCD-32g58 SLP subunits in the samples (Fig. 2c, left panel). Moreover, a Western blot performed against transferred V_H_Hs confirmed all V_H_Hs had their His_6_ tag. The V_H_Hs SLP_V_H_H5, SLP_V_H_H12, SLP_V_H_H23, SLP_V_H_H46, and SLP_V_H_H49 bound the LMW subunit of QCD-32g58 SLP, consistent with our SPR results (Fig. 2b), and indicating that these V_H_Hs recognized a linear epitope. The remaining V_H_Hs were weakly positive, or negative altogether, by Western blot for binding to the LMW subunit of QCD-32g58 SLP, indicating that they may recognize conformational epitopes, or have too low of an affinity and/or *k*_off_s too rapid to produce a detectible signal.

### Binding of V_H_Hs to *C. difficile* cells

**Fig. 3:**
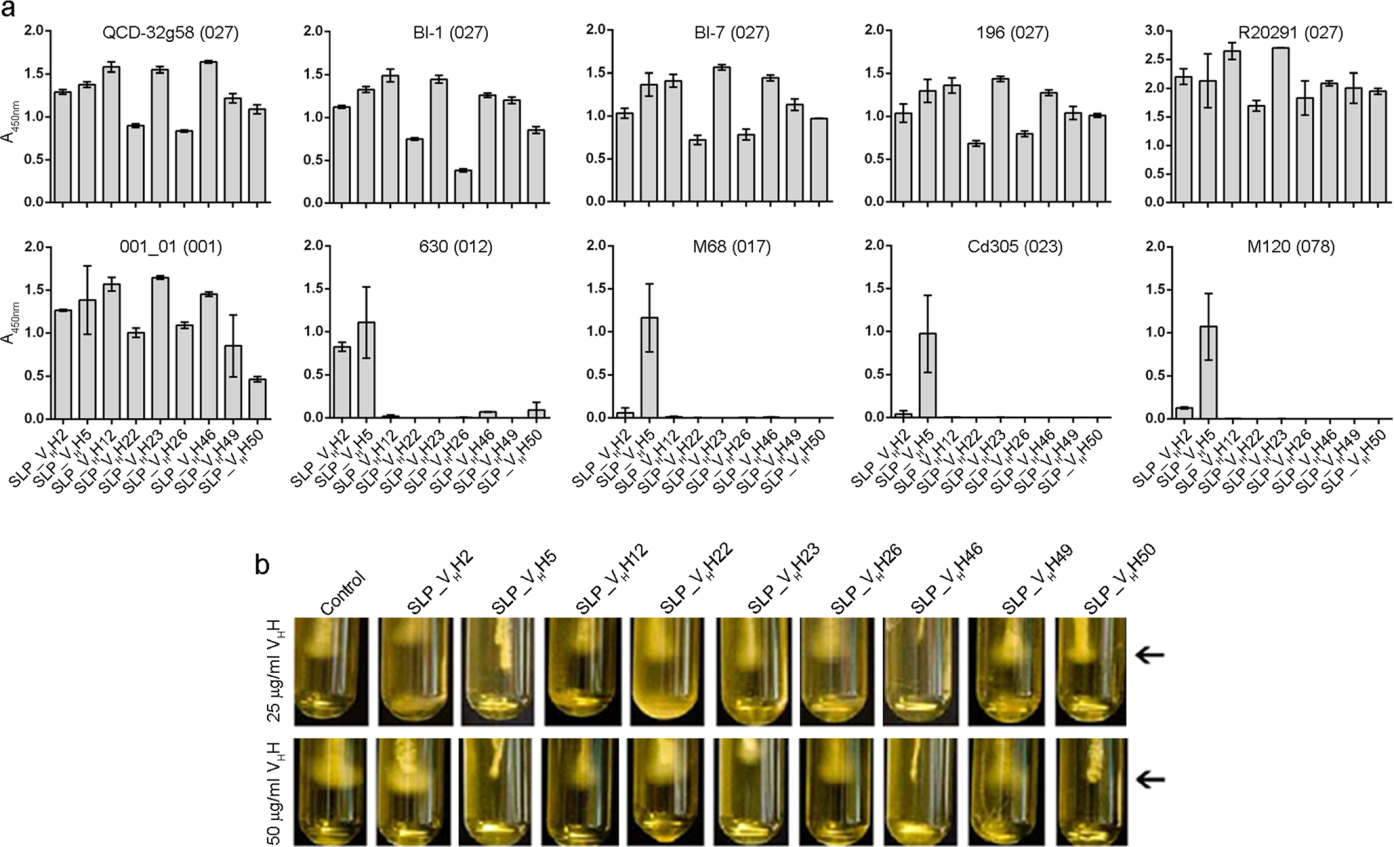
SLP-specific V_H_Hs bind *C. difficile* cells and inhibit motility. **a** Whole cell ELISA demonstrating the binding of V_H_Hs to various *C. difficile* strains. **b** *C. difficile* (QCD-32g58) stabs after 23 h comparing the effects of 25 and 50 μg/ml V_H_H concentrations on bacterial motility. SLP_V_H_H5, SLP_V_H_H46, and SLP_V_H_H50 showed inhibition of *C. difficile* motility, denoted with *arrows* at the tip of the stabs

ELISA was used to determine the ability of each V_H_H to bind to a number of *C. difficile* clinical isolates. All SLP-specific V_H_Hs in this study bound bacterial cells of strain QCD-32g58 (Fig. 3a). In addition, strong reactivity of each V_H_H to the bacterial cell surface of a number of other *C. difficile* isolates which belong to the same 027 hypervirulent ribotype (BI-1, BI-7, 196, R20291) as well as ribotype 001 (strain 001_01) was observed. In contrast, V_H_H reactivity to the cell surface of representative strains from other ribotypes (012, 017, 023, and 078) was far more restricted, suggesting considerable diversity in the LMW SLP epitopes displayed among distinct lineages of *C. difficile*. Interestingly, SLP_V_H_H5 was able to recognize all *C. difficile* isolates tested, representing a number of distinct ribotypes. While both SLP_V_H_H2 and SLP_V_H_H26 were shown to cross-react to 630 SLP in phage ELISA and SPR assays, it was only SLP_V_H_H2 that cross-reacted to 630 SLP in cell binding assays.

### *C. difficile* motility assays

Despite a lack of evidence in the literature relating SLP function to bacterial motility, we nonetheless sought to test the ability of SLP-specific V_H_Hs to inhibit *C. difficile* (QCD-32g58 strain) motility. Culture tubes containing BHI-agar supplemented with V_H_Hs at either 25 μg/ml (~1.5 μM) or 50 μg/ml (~3 μM) were inoculated with stabs of *C. difficile* and cultured for 23 h. Growth was monitored and photographed 23 h postinoculation (Fig. 3b). Motile cells displayed a diffuse spreading flare of growth at the bottom of the inoculating stab. The results demonstrated that at 23 h postinoculation using 25-μg/ml antibody concentrations, SLP_V_H_H5 and SLP_V_H_H46 completely inhibited *C. difficile* motility. SLP_V_H_H50 showed slight inhibition of motility at 25 μg/ml. The remaining V_H_Hs did not inhibit motility at concentrations of 25 μg/ml. To test whether motility inhibition was concentration dependent, we doubled the antibody concentration to 50 μg/ml (Fig. 3b). Similar to the lower concentration, SLP_V_H_H5 and SLP_V_H_H46 clearly inhibited *C. difficile* motility. Increasing the concentration of SLP_V_H_H50 to 50 μg/ml resulted in complete inhibition of *C. difficile* motility.

## Discussion

The outer surface of many bacteria is covered in a proteinaceous coat called the S-layer (surface layer) that is involved in growth, function, and interaction with the host (Fagan and Fairweather 2014). In Gram-positive species, such as *C. difficile*, SLPs have been shown to play a role in adherence to gastrointestinal tract cells and extracellular matrix components (Calabi et al. 2002; Takumi et al. 1991), and recently, SLPs were shown to have a role in activating innate and adaptive immunity through TLR4 (Ryan et al. 2011) and induce pro-inflammatory cytokines (Bianco et al. 2011; Collins et al. 2014). It has been known for several years that patients with recurrent episodes of *C. difficile* have significantly lower anti-SLP IgM titers than patients experiencing a single episode of *C. difficile* infection (Drudy et al. 2004). In addition, active immunization of hamsters with SLPs elucidated partial protection when challenged with *C. difficile* (Ni Eidhin et al. 2008). Collectively, this suggests that SLPs may have a critical role in *C. difficile* pathogenesis and virulence in humans, making them targets for diagnostic probes, vaccine development and novel therapeutic agents. In *C. difficile*, mature SLPs consist of HMW and LMW subunits which are produced by proteolytic cleavage of a single polypeptide chain (SlpA). In a mature SLP, the LMW subunit is displayed toward the environment and shows higher sequence variability than the HMW subunit (Calabi and Fairweather 2002; Merrigan et al. 2013).

To explore the use of antibodies targeting novel *C. difficile* virulence factors, we produced high-affinity llama V_H_Hs to *C. difficile* SLPs. We isolated SLPs from the hypervirulent QCD-32g58 strain (027 ribotype) and the 630 reference strain (012 ribotype), immunized a llama with both simultaneously, isolated several V_H_Hs, and characterized these antibodies. Immunization with SLPs generated a strong heavy-chain antibody immune response in the llama, indicating the SLPs were very immunogenic. From a phage display library panned with SLPs from QCD-32g58, nine unique V_H_Hs were isolated. By phage ELISA and SPR, all recognized QCD-32g58 SLP, while two (SLP_V_H_H2 and SLP_V_H_H26) cross-reacted to 630 SLP, with at least more than half of the V_H_Hs recognizing linear epitopes. SPR binding of V_H_Hs revealed high-affinity binding to QCD-32g58 SLP with *K*_D_s as low as 3–6 nM, but nonetheless, several V_H_Hs also had significantly higher *K*_D_s, as high as 580 nM, a *K*_D_ range pattern frequently seen with V_H_Hs obtained from immune V_H_H phage display libraries. Interestingly, the four V_H_Hs with the highest affinities (3–6 nM) all recognize linear epitopes. Despite immunizing and panning with the QCD-32g58 whole SLP, all of the V_H_Hs targeted the highly variable LMW subunit. The HMW subunit is conserved across *C. difficile* isolates and the LMW subunit is considerably more variable (Calabi and Fairweather 2002; Merrigan et al. 2013). In agreement with our findings, between the LMW and HMW subunits, the LMW one has been shown to be the immunodominant antigen elsewhere (Ausiello et al. 2006; Péchiné et al. 2007).

With respect to thermostability, V_H_Hs showed *T*_m_s as high as 75 °C, although engineered V_H_Hs with higher *T*_m_s have been previously reported, in the range of 79–94 °C (Hussack et al. 2011b; Zabetakis et al. 2014). V_H_Hs also showed significant resistance to the GI enzyme pepsin with two V_H_Hs having pepsin resistance as high as 20 % at a physiologically relevant pepsin concentration (100 μg/ml). Noticeably, three out of the four V_H_Hs that showed pepsin resistance at a relatively high enzyme concentration (50 μg/ml) have the highest *T*_m_s (73.7–75.4 °C), and SLP_V_H_H22, which was the most resistant V_H_H, had a pair of Cys at positions 55 and in CDR3 that formed an extra disulfide linkage. Previously, a positive correlation was found between pepsin resistance and *T*_m_, and mutations that increased *T*_m_ also increased pepsin resistance (Hussack et al. 2011b). The extra noncanonical disulfide linkage in SLP_V_H_H22 may be a contributor to its high *T*_m_ and/or pepsin resistance. Previously, similar noncanonical (inter-CDR1-CDR3; inter-CDR2-CDR3) disulfide linkages were shown to increase the stability of V_H_Hs (Govaert et al. 2012; Zabetakis et al. 2014). In particular, a disulfide linkage formed between a pair of Cys residues at positions 55 and in CDR3 improved the *T*_m_ of a V_H_H by several degrees (Zabetakis et al. 2014). However, we find that in addition to forming the expected noncanonical disulfide linkage between them, Cys55 and CDR3 Cys also pair up with Cys23 or Cys104—which are involved in a highly conserved canonical disulfide linkage in V_H_Hs—to form unusual disulfide linkages not reported previously. Whether these unusual disulfide linkages are the result of heterologous expression in *E. coli* is not clear to us. It is also unclear if they are present in significant proportions of the V_H_H population.

We tested the ability of V_H_Hs to bind *C. difficile* whole cells in ELISA, which presents the SLP protein in a more natural context for antibody binding. All nine V_H_Hs bound QCD-32g58 cells and, not surprisingly, all other 027 ribotype strains tested, including BI-1, BI-7, 196, and R20291, which have identical LMW subunit SLP sequences to QCD-32g58. These results confirm the feasibility of using purified, out-of-natural-context SLP as an immunogen and target antigen for panning experiments for obtaining anti-SLP antibodies that recognize parent cells equally well. As well, the panel of V_H_Hs all bound to a 001 ribotype strain, indicating that at least the LMW subunit of 001 ribotype strain should have high sequence identity to the SLP LMW subunits from the aforementioned 027 ribotypes. SLP_V_H_H2 showed binding to 630, which was expected given the evidence of cross-reactivity in ELISA and SPR. SLP_V_H_H26 did not show binding to 630 cells, despite earlier ELISA and SPR evidence showing binding to 630 SLPs. Interestingly, SLP_V_H_H5 bound all ribotypes tested in the cell ELISA format, indicating the antibody is broadly cross-reactive. Why SLP_V_H_H5 failed to recognize 630 SLPs in phage ELISA and SPR is not entirely clear, but it could be due to the fact that immobilizing the SLP prevented antibody binding by masking or changing the conformation of the epitope. Differential epitope presentations may also account for binding inconsistencies observed for SLP_V_H_H26 between phage ELISA/SPR assays and cell ELISA assay. The remaining V_H_Hs did not bind cells representative of 012, 017, 023 or 078 ribotypes. The low frequency of cross-reactive V_H_Hs may not be surprising given the low amino acid identity among SLP LMW subunits from different ribotypes. We speculate that at least six different epitopes are being recognized by our pool of V_H_Hs, given that there are five different specificities inferred from cell binding, motility and ELISA/SPR assays, one represented by SLP_V_H_H2, one by SLP_V_H_H5, one by SLP_V_H_H26 that cross-reacted to 630 strain in phage ELISA/SPR, one by SLP_V_H_H46 and SLP_V_H_H50 that inhibited motility, and one represented by the remaining V_H_Hs (SLP_V_H_H12, SLP_V_H_H22, SLP_V_H_H23, and SLP_V_H_H49). This latter group can be divided into those binding a linear epitope and those binding a conformational epitope as determined by Western blotting.

Despite their variability, alignment of LMW SLP amino acid sequences from several *C. difficile* ribotypes reveal stretches of conserved residues that could represent epitopes for cross-reactive antibody binding (Fig. S4). Specifically, residues 8–11, 72–83, 249–261, 264–275, and 299–321, numbered based on the 630 sequence, show significant homology across all aligned ribotypes (Fagan et al. 2009). Based on LMW SLP structural data, the LMW SLP is composed of domain 1 (residues 1–87 and residues 242–248) and domain 2 (residues 97–233), with domain 1 facing toward the bacterial cell wall and the HMW subunit, while domain 2 is orientated away, toward the environment (Fagan et al. 2009). The residues of domain 2 show the most variability among ribotypes (Fig. S4) and are also likely the most accessible for antibody binding given they extend away from the bacterial surface. In the case of the broadly cross-reactive SLP_V_H_H5 antibody, it is possible that even though domain 1 of the LMW SLP faces inward toward the cell wall and is in close proximity to the HMW SLP interaction domain, domain 1 residues remain accessible for binding. Further studies on this antibody, including co-crystallization structure determination, could reveal the true nature of the LMW epitope.

Somewhat surprisingly, in agar-stab motility assays, several V_H_Hs were capable of inhibiting motility of QCD-32g58 cells. In particular, SLP_V_H_H5 and SLP_V_H_H46 were capable of inhibiting motility at both high and low antibody concentrations. To a lesser degree, SLP_V_H_H50 was also found to inhibit motility. Higher affinity, faster *k*_on_/slower *k*_off_ and/or the nature of epitope of SLP_V_H_H5 and SLP_V_H_H46 may be responsible for their greater motility inhibition potency compared to SLP_V_H_H50 (based on Western blot and cell-binding experiments, SLP_V_H_H5 and SLP_V_H_H46 have different epitopes than SLP_V_H_H50). There are a limited number of reports of polyclonal antibody and mAb preparations targeting *C. difficile* SLPs; however, none have examined the ability of antibodies to inhibit *C. difficile* motility. Takumi et al. (1991) produced anti-SLP Fab fragments and used them to inhibit the adherence of *C. difficile* to human cervical cancer cells and mouse fibroblast cells. O’Brien et al. (2005) showed that the injection of hamsters with antibodies to SLPs prolonged the survival of *C. difficile*-infected hamsters. More recently, anti-HMW SlpA and anti-LMW SlpA polyclonal antiserum was shown to reduce *C. difficile* strain 630 adherence to C2_BBE_ human colonic epithelial cells although the precise mechanism was not defined (Merrigan et al. 2013). While our study is unique in that we appear to inhibit motility through targeting *C. difficile* SLPs, others have found motility-inhibiting affinity reagents by targeting an alternative bacterial cell surface structure, namely the lipopolysaccharide (LPS). A mAb that bound the LPS of *Salmonella enterica* was shown to inhibit flagellum-based motility (Forbes et al. 2008). Similarly, P22sTsp, a phage tailspike protein that binds to LPS was also able to inhibit the motility of *Salmonella enterica* serovar Typhimurium (Waseh et al. 2010). As would be expected an anti-flagellin mAb inhibited the motility of multi-drug resistant *Pseudomonas aeruginosa* and curbed lethality in mice (Adawi et al. 2012). In another study, anti-*P. aeruginosa* flagellin V_H_Hs inhibited the motility and biofilm formation of *P. aeruginosa* (Adams et al. 2014). Similarly an anti-*Campylobacter jejuni* flagellin V_H_H inhibited the motility of *C. jejuni* (Hussack et al. 2014; Riazi et al. 2013). To date, there is no known report of SLP interactions with motility factors in *C. difficile* and SLPs remain the primary adherence factors of *C. difficile*. However, the theme of blocking a surface antigen which is high in abundance, wherein motility is reduced, is presented in this study and warrants further investigation. Our data suggests that antibodies binding to *C. difficile* SLPs may provide some form of steric hindrance to the effective functioning of the flagellar motility apparatus. Continued studies on the structure and function of *C. difficile* SLPs and their role in host-pathogen interactions, as well as nature of the LMW epitope recognized by broadly cross-reactive SLP antibodies which inhibit motility, will help in elucidating this unusual interaction between two key surface structures. Whether our SLP-specific V_H_Hs interfere with cell growth and biofilm formation warrants further investigation.

In conclusion, we have isolated a panel of high-affinity V_H_Hs that target the LMW SLP subunit of *C. difficile* QCD-32g58. Many of the V_H_Hs recognized several strains within the 027 ribotype, which is the predominant hypervirulent ribotype seen in hospital-acquired (nosocomial) *C. difficile* infections. One V_H_H (SLP_V_H_H5) additionally recognized two strains from ribotypes 017 and 078 which are recognized as emerging PCR ribotypes implicated in recent outbreaks with increased disease severity (Cheknis et al. 2009; Hunt and Ballard 2013). Of additional significance, a subset of four V_H_Hs (SLP_V_H_H5, SLP_V_H_H12, SLP_V_H_H23, and SLP_V_H_H46) possessed high affinities, a similar set (SLP_V_H_H5, SLP_V_H_H46, and SLP_V_H_H50) inhibited motility and two (SLP_V_H_H12 and SLP_V_H_H23) demonstrated strong resistance to the GI protease pepsin. Affinity maturation combined with a disulfide engineering approach described previously (Hussack et al. 2011b; Hussack et al. 2014; Saerens et al. 2008) can be employed to further increase their affinities, motility inhibition capability and resistance to GI proteases, making them suitable oral/GI therapeutics against CDAD or useful agents in the validation of SLP as a vaccine target. A combination therapy approach involving the present anti-SLP V_H_Hs and previously described toxin A- and toxin B-specific V_H_Hs (Hussack et al. 2011a; Yang et al. 2014) also appears attractive.

## Electronic supplementary material

ESM 1 (PDF 1074 kb)
