# Supplementary material for: Targeting surface-layer proteins with single-domain antibodies: a potential therapeutic approach against Clostridium difficile-associated disease
Source: Appl Microbiol Biotechnol. 2015 May 5;99:8549–62. doi: 10.1007/s00253-015-6594-1 (PMC4768215; doi:10.1007/s00253-015-6594-1)
Supplement: Supplementary file 1 — (PDF 1074 kb) [file 253_2015_6594_MOESM1_ESM.pdf]

Supplementary Material

Applied Microbiology and Biotechnology

Targeting surface-layer proteins with single-domain antibodies: a potential therapeutic approach against *C. difficile*-associated disease

Hiba Kandalaft[1], Greg Hussack[1], Annie Aubry[1], Henk van Faassen[1], Yonghong Guan[1], Mehdi Arbabi-Ghahroudi[1,2,3], Roger MacKenzie[1,3], Susan M Logan[1,4], Jamshid Tanha[1,3,4,5]

[1]Human Health Therapeutics Portfolio, National Research Council Canada, Ottawa, ON, Canada K1A 0R6
[2]Department of Biology, Carleton University, Ottawa, ON, Canada K1S 5B6
[3]School of Environmental Sciences, University of Guelph, Guelph, ON, Canada N1G 2W1,
[4]Department of Biochemistry, Microbiology and Immunology, University of Ottawa, Ottawa, ON, Canada K1N 6N5
[5]Corresponding author: Jamshid Tanha; Affiliation: Human Health Therapeutics Portfolio, National Research Council Canada, ON, Canada K1A 0R6; E-mail address: Jamshid.Tanha@nrc-cnrc.gc.ca

Table S1. Disulfide bond mapping of SLP_V_HH22 and SLP_V_HH50.

V_HH	Tryptic peptides[a]	M_{for} (Da)[b]	M_{exp} (Da)[b]	ΔM (Da)[b]	Disulfide bond
SLP_V_HH22	**C**SSLDMALGALATR<br>EHEGIS**C**ISSNGGSTYYTDSVK	3738.68	3739.17	-0.49	55-CDR3
	**C**SSLDMALGALATR<br>LS**C**AASGFTLDSYAIGWFR	3469.64	3469.54	0.1	23-CDR3
	LS**C**AASGFTLDSYAIGWFR<br>EHEGIS**C**ISSNGGSTYYTDSVK	4394.97	4395.55	-0.58	23-55
SLP_V_HH50	EGVA**C**ISSDDR<br>G**C**APGHDDYWGQGTQVTVSSGSEQK	3741.59	3741.20	0.39	55-CDR3
	EGVA**C**ISSDDR<br>NTVYLQMNNLKPEDTAVYY**C**ASK	3812.73	3812.63	0.1	55-104

[a]Major trypsin-cleaved peptides containing disulfide linkages (lines connecting the cysteine residues) are shown. ΔM = M_{for} - M_{exp}. [b]The very close match between M_{for} (formula molecular mass) and M_{exp} (experimental molecular mass) reflected in marginal ΔM values indicates the presence of the shown disulfide bonds.

```
630        MNKKNIAIAMSGLTVLASAAPVFAAT------TGTQGYTVVKNDWKKAVKQLQDGLKDNS
QCD-32g58  MNKKNIAIAMSGLTVLASAAPVFAAEDMSKVETGDQGYTVVQSKYKKAVEQLQKGLLDGS
           *************************       ** ******:..:****:***.** *.*

630        IGKITVSFNDGVVGEVAPKSANKKADRDAAAEKLYNLVNTQLDKLGDGDYVDFSVDYNLE
QCD-32g58  ITEIKIFFEGTLASTIK---VGAELSAEDASKLLFTQVDNKLDNLGDGDYVDFLISSPAE
           * :*.: *:. :.. :    .. : . : *:: *:. *:.:**:********* :.   *

630        NKIITNQADAEAIVTKLNSLNEKTLIDIATKDTFGMVSKTQDSEGKNVAATKALKVKDVA
QCD-32g58  GDKVT----TSKLVALKNLTGGTSAIKVATSSIIGEVENAGTPGAKNTAPSSAAVMSMSD
           .. :*    :. :*:  *  . .: *.:**.. :* *.::  . .**.*.:.*  :.

630        TFGLKSGGSEDTGYVVEMKAGAVEDKYGKVGDSTAGIAINLPSTGLEYAGKGTTIDFNKT
QCD-32g58  VFDTAFTDSTETAVKLTIKDAMKTKKFGLVDGTTYSTGLQFADGKTEKIVKLGDSDTINL
           .*.    .* :*.  : :* .   .*:* *..:* . .::..    *   *    *  :

630        LKVDVTGGSTPSAVAVSGFVTKDDTDLAKSGTINVRVINAKEESIDIDASSYTSAENLAK
QCD-32g58  AKELIITPASANDQAATIEFAKPTTQSGSPVITKLRILNAKEETIDIDASSSKTAQDLAK
            *  :   ::..  *.:  .:*  *: ...   ::*::*****:******* .:*::***
                                                                  Cwp84
630        RYVFDPDEISEAYKAIVALQNDGIESNLVQLVNGKYQVIFYPEGKRLETKSAN-DTIASQ
QCD-32g58  KYVFNKTDLNTLYRVLNGDEAD--TNRLVEEVSGKYQVVLYPEGKRVTTKSAAKASIADE
           :***:  ::.  *:.: . : *   ..**: *.*****::******: ****   :**.:

630        DTPAKVVIKANKLKDLKDYVDDLKTYNNTYSNVVTVAGEDRIETAIELSSKYYNSDDKNA
QCD-32g58  NSPVKLTLKSDKKKDLKDYVDDLRTYNNGYSNAIEVAGEDRIETAIALSQKYYNSDDENA
           ::*.*:.:*::* **********:**** ***.: *********** **.*******:**

630        ITDKAVNDIVLVGSTSIVDGLVASPLASEKTAPLLLTSKDKLDSSVKSEIKRVMNLKSDT
QCD-32g58  IFRDSVDNVVLVGGNAIVDGLVASPLASEKKAPLLLTSKDKLDSSVKAEIKRVMNIKSTT
           *  .:*:::****..:**************.****************:*******:** *

630        GINTSKKVYLAGGVNSISKDVENELKNMGLKVTRLSGEDRYETSLAIADEIGLDNDKAFV
QCD-32g58  GINTSKKVYLAGGVNSISKEVENELKDMGLKVTRLAGDDRYETSLKIADEVGLDNDKAFV
           *******************:******:********:*:******* ****:*********

630        VGGTGLADAMSIAPVASQLK---------DGDATPIVVVDGKAKEISDDAKSFLGTSDVD
QCD-32g58  VGGTGLADAMSIAPVASQLRNANGKMDLADGDATPIVVVDGKAKTINDDVKDFLDDSQVD
           *******************:         *************** *.**.*.**. *:**

630        IIGGKNSVSKEIEESIDSATGKTPDRISGDDRQATNAEVLKEDDYFTD-----GEVVNYF
QCD-32g58  IIGGENSVSKDVENAIDDATGKSPDRYSGDDRQATNAKVIKESSYYQDNLNNDKKVVNFF
           ****:*****::*::**.****:*** **********:*:**..*: *      :***:*

630        VAKDGSTKEDQLVDALAAAPIAGRFKE---------------------------SPAPII
QCD-32g58  VAKDGSTKEDQLVDALAAAPVAANFGVTLNSDGKPVDKDGKVLTGSDNDKNKLVSPAPIV
           ********************:*..*                             *****:

630        LATDTLSSDQNVAVSKAVPKDGGTNLVQVGKGIASSVINKMKDLLDM
QCD-32g58  LATDSLSSDQSVSISKVLDKDNGENLVQVGKGIATSVINKLKDLLSM
           ****:*****.*::**.: **.* **********:*****:****.*
```

Fig. S1 Amino acid sequence alignment of SLPs from *C. difficile* strains 630 and QCD-32g58. The proteins were aligned using Clustal Omega. Indicated are the SLP signal-sequence (underlined), signal sequence cleavage site (clear triangle), LMW SLP (highlighted grey), Cwp84 protease cleavage site (solid triangle) and HMW SLP (italicized)

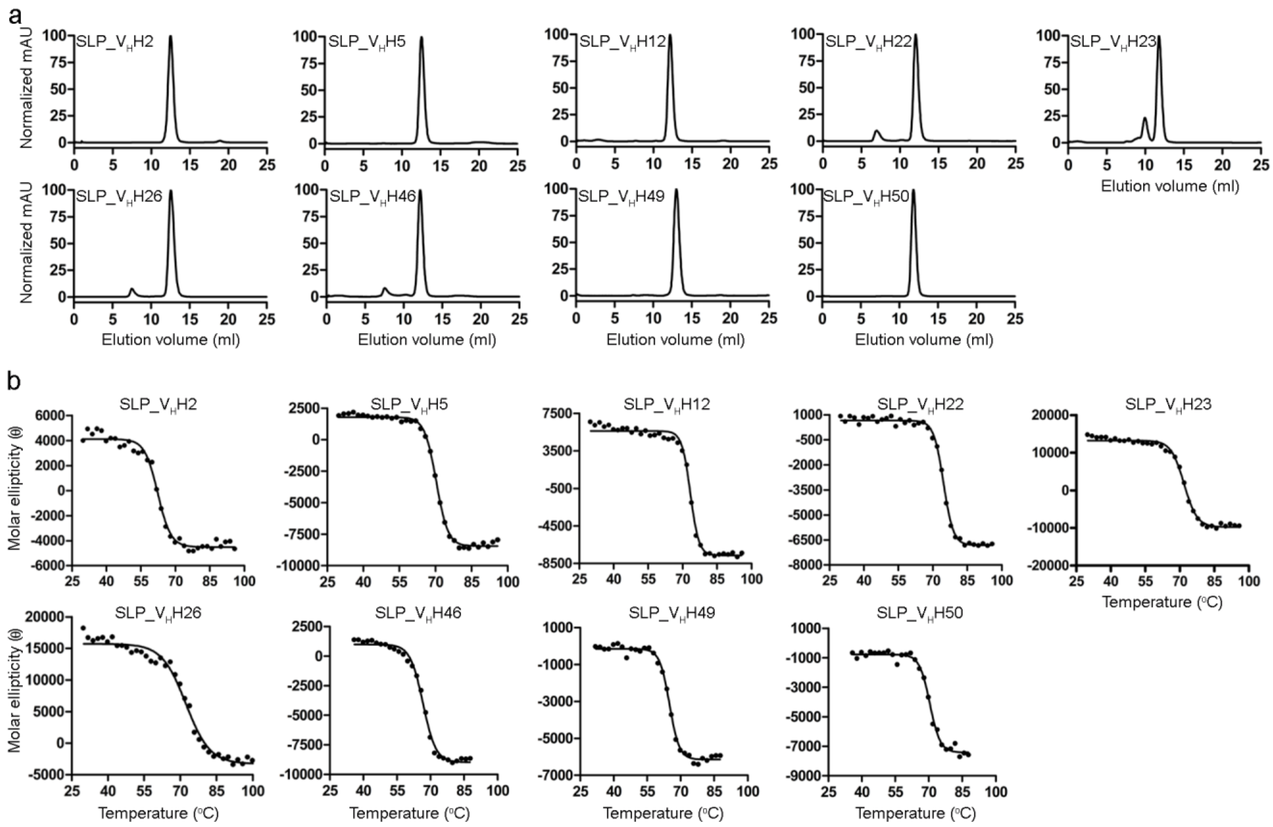

Fig. S2 Biophysical characterization of SLP-binding V_HHs. **a** SEC Superdex^{TM} 75 profiles. Elution volume values of V_HHs were used to calculate their apparent molecular masses (M_{app}s) from a protein standards curve. The M_{app}s are recorded in Table 1. **b** Thermal unfolding (T_m) curves.

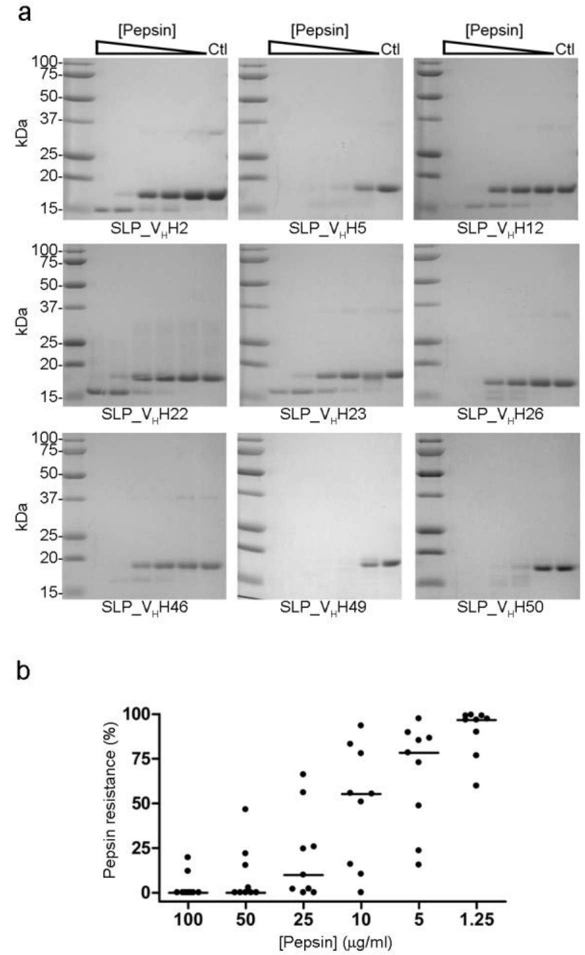

Fig. S3 Pepsin digestion assays. **a** SDS-PAGE gel analysis of V_HHs digested with pepsin at concentrations ranging from 50 µg/ml to 1.25 µg/ml. Undigested V_HHs served as a control (Ctl) for densitometry analysis. The first lanes on the left side of each gel represent molecular mass markers. **b** Summary plot of V_HH resistance over various pepsin concentrations, including digestion of V_HHs with 100 µg/ml pepsin not shown in **a**. Bars represent the median pepsin resistance at a given pepsin concentration

```
          1                        20                  40
630       AT------TGTQGYTVVKNDWKKAVKQLQDGLKDNSIGKITVSFNDGVVGEVAPKSA---
M68       DS------T-TPGYTVVKNDWKKAVKQLQDGLKNKTISTIKVSFNGNSVGEVTPASS---
QCD       AEDMSKVETGDQGYTVVQSKYKKAVEQLQKGLLDGSITEIKIFFEGTLASTIKVGAE---
M120      AD---EQVKYQNTYTVVQSKYEKALKDMQKGITDKKIKSIAISYEGKPVTTITVADMDTK
                  .    ****:..::**::::*.*: : .*  * :  ::  .  :

                   60                  80                  100
630       -NKKADRDAAAEKLYNLVNTQLDKLGDGDYVDFSVDYNLENKIITNQADAEAIVTKLNSL
M68       GAKKADRDAAAEKLYNLVNTQLDKLGDGDYVDFEVTYNLATQIITK-AEAEAVLTKLQQY
QCD       ----LSAEDASKLLFTQVDNKLDNLGDGDYVDFLISSPAEGDKVTTSKL-V-ALKNLTGG
M120      GKTSTKEELASALLKTTVNDKLDNLGDGDYVDFDITYVGDADRLTAGDL-N-TFAKGIAD
               . : *:  * . *:  :**:********* :      . :*        . :

                   120                 140                 160
630       NEKTLIDIATKDTFGMVSKTQDSEGKNVAATKALKVKDVATFGLKSGGSED-TGYVVEMK
M68       NDKVLINSATDTVKGMVSDTQVDSK-N-VAANPLKVSDMYTIPSAITGSDD-SGYSIAKP
QCD       TSAIK--VATSSIIGEVENAGTPGAKNTAPSSAAVMSMSDVFDTAFTDST-ETAVKLTIK
M120      STEKKIPAAKGSNYGVAKTNSGTGKLT--TDTEAVIS------TSIEGKVEGNNLTISLK
          .       *.    * ..        .    .   :.           .   .   :

                     180                     200                 220
630       A-GAVEDKYGKVGDST---AGIAINLPST-GLEYAGKGTTIDFNKTLKVDVTGGSTPSAV
M68       TEKTTSLLYGTVGDAT---AGKAITVDTASNEAFAGNGKVIDYNKSFKATVQGDGT---V
QCD       -DAMKTKKFGLVDGTT-YSTGLQFADGKTEKIVKLGDSDTINLAKELIITPASANDQAAT
M120      -DA--PSKVGVIGANNDTLADVTFADDA-KLTVSVGD-PKIDLAKSFIFDTKTGKL-GGI
                   * :   .   :   :           *.   *:  * :

                         240                 260                 280
630       AVSGFVTKDDTDLAKSGTINVRVINAKEESIDIDASSYTSAENLAKRYVFDPDEISEAYK
M68       KTSGVVLKDASDMAATGTIKVRVTSAKEESIDVDSSSYISAENLAKKYVFNPKEVSEAYN
QCD       I---EFAKPTTQSGSPVITKLRILNAKEETIDIDASSSKTAQDLAKKYVFNKTDLNTLYR
M120      V--------EKENDATEHAYVRVINAKEQTIDLDASSYKSAEDLAKAYAFDVNELKTLYT
                    .:        :*: .***::**:*:**  :*::*** *.*:  ::.  *

                          300                 320
630       AIVALQNDGI-ESNLVQLVNGKYQVIFYPEGKRLETKS
M68       AIVALQNDGI-ESDLVQLVNGKYQVIFYPEGKRLETKS
QCD       VLNGDEADTN---RLVEEVSGKYQVVLYPEGKRVTTKS
M120      EIEAYQKDSNNKTDKVQIVDGKYQTILYAEGKRLTTKS
           : . : *       *: *.****.::* ****: ***
```

Fig. S4 Amino acid sequence alignment of LMW SLPs from *C. difficile* strains representative of 012 ribotype (630), 017 ribotype (M68), 027 ribotype (QCD-32g58; “QCD”) and 078 ribotype (M120). The proteins were aligned using Clustal Omega and numbered according to the 630 sequence (Fagan et al. 2009). Areas showing a high degree of sequence homology are shaded in grey. The dark line from residue 260 to residue 321 indicates the region of the LMW SLP that interacts with the HMW SLP

References

Fagan RP, Albesa-Jove D, Qazi O, Svergun DI, Brown KA, Fairweather NF (2009) Structural insights into the molecular organization of the S-layer from *Clostridium difficile*. Mol Microbiol 71:1308-1322
